# PROTECTING RETAIL CUSTOMERS FROM GIFT CARD PAYMENT SCAMS

**DOI:** 10.1093/geroni/igac059.2153

**Published:** 2022-12-20

**Authors:** Dionne Bailey, Marti DeLiema

**Affiliations:** University of Minnesota, Minneapolis, Minnesota, United States; University of Minnesota - Twin Cities, Saint Paul, Minnesota, United States

## Abstract

Older adults are frequently targeted by scams and fraud, a persistent and growing problem in the U.S. According to the Federal Trade Commission, gift cards were the most common method of payment requested by fraud criminals in 2021. The purpose of this AARP-sponsored study is to investigate gift card payment scams by analyzing the retail sector for current interventions and opportunities to protect customers from fraud. Through secret shopping at 23 retail locations in a large U.S. city, we documented the existing status of merchant controls designed to prevent suspicious gift card purchases, as well as how these measures work in practice. At each location, the "secret shopper" attempted to purchase between $400-$1000 in gift cards from a sales clerk, while another researcher stood in line nearby to observe the interaction. ​​Nearly 80% of retail stores had signs warning customers about gift card frauds. Fewer than 50% imposed limits on the purchase amount; more than 40% required manager approval, and five of the transactions resulted in manager involvement. Current retail measures would only limit purchases in around 22% of gift card frauds and would completely halt about 9% of transactions. Results indicate that despite warnings, retailers are not doing enough to protect customers from fraud. New policies are needed to advocate for reduced gift card purchase limits, increase public awareness, educate retail employees on the red flags, and enforce anti-money laundering rules to limit fraud.

